# Preference and Performance in Plant–Herbivore Interactions across Latitude–A Study in U.S. Atlantic Salt Marshes

**DOI:** 10.1371/journal.pone.0059829

**Published:** 2013-03-22

**Authors:** Chuan-Kai Ho, Steven C. Pennings

**Affiliations:** 1 Institute of Ecology and Evolutionary Biology, National Taiwan University, Taipei, Taiwan; 2 Department of Life Science, National Taiwan University, Taipei, Taiwan; 3 Department of Biology and Biochemistry, University of Houston, Houston, Texas, United States of America; University of Alberta, Canada

## Abstract

High-latitude plants are often more palatable to herbivores than low-latitude conspecifics. Does increased plant palatability lead to better herbivore performance? Our field and laboratory work investigated (A) whether high-latitude plants have traits indicating that they should be higher-quality foods for herbivores; (B) whether geographic differences in plant quality are more important than local adaptation of herbivores. We studied 3 plant species and 6 invertebrate herbivores in U.S. Atlantic Coast. Past studies had shown high-latitude individuals of these plants are more palatable than low-latitude conspecifics. We documented plant traits and herbivore performance (body size) in the field across latitude. We collected individuals from different latitudes for factorial (plant region x herbivore region) laboratory experiments, examining how herbivore performance was affected by plant region, herbivore region, and their interaction (i.e., local adaptation). Field surveys suggested high-latitude plants were likely of higher quality to herbivores. Leaf nitrogen content in all plant species increased toward high latitudes, consistent with lower leaf C/N and higher leaf chlorophyll content at high latitudes. Furthermore, leaf toughness decreased toward higher latitudes in 1 species. The body size of 4 herbivore species increased with latitude, consistent with high-latitude leaves being of higher quality, while 2 grasshopper species showed the opposite pattern, likely due to life-history constraints. In the laboratory, high-latitude plants supported better performance in 4 herbivore species (marginal in the 5th). The geographic region where herbivores were collected affected herbivore performance in all 6 species; however, the pattern was mixed, indicating a lack of local adaptation by herbivores to plants from their own geographic region. Our results suggest that more-palatable plants at high latitudes support better herbivore growth. Given that geographic origin of either plants or herbivores can affect herbivore performance, the nature of plant-herbivore interactions is likely to change if climate change “reshuffles” plant and herbivore populations across latitude.

## Introduction

Ecologists have long been interested in latitudinal gradients in diversity and species interactions because these are likely to reveal fundamental effects of geography and climate on ecological processes [1], [2], [3], [4]. Given the unprecedented speed of current global environmental changes [5], research in the field of biogeography is particularly timely because it can lend essential insights into likely effects of environmental change on communities and ecological processes.

**Figure 1:**
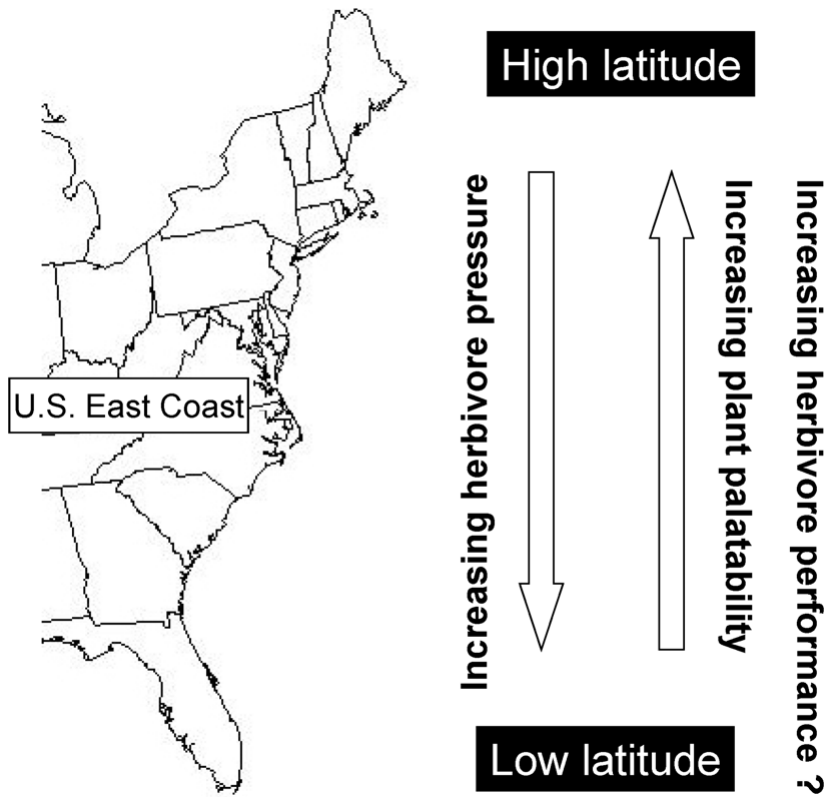
Known and hypothesized plant-herbivore interactions across latitude in salt marshes on the U.S. Atlantic Coast. This study tested if increased plant palatability predicts better herbivore performance at high latitudes.

One paradigm of biogeography is that predation and herbivory are more intense, and prey defenses better developed, in the tropics than in the temperate zone [1], [4], [6], [7], [8], [9]. In the case of plant-herbivore interactions, this theory predicts higher plant palatability, due to weaker defenses against herbivores, at high versus low latitudes (Fig. 1). The most extensive tests of this theory come from coastal salt marshes, systems with relatively few plant species that have large geographic distributions, allowing intraspecific or intrageneric comparisons across a wide range of latitude. A community-wide study in salt marshes on the U.S. Atlantic Coast found that herbivores (13 species) preferred to eat plants (10 species) from high versus low latitudes, and this result occurred regardless of the geographic location of the assay, herbivore species, year, or season of plant collection [9]. A similar test in European salt marshes found parallel results [10]. Common-garden greenhouse studies have shown that latitudinal variation in plant palatability and relevant traits were in part constitutive and continued to exist over several generations in the laboratory [11]. Comparisons of herbivore density and herbivore damage to leaves found that both were greater at low latitudes, suggesting that selection driven by herbivore pressure might help explain differences in plant palatability across latitude [12].

Although a growing number of studies have shown that plant traits related to palatability vary across latitude [13], [14], [15], [16], [17], it has not been determined whether geographic differences in plant traits or palatability actually reflect differences in plant quality. In short, do latitudinal differences in plant palatability predict latitudinal differences in the performance of herbivores eating these plants (Fig. 1)? Many studies have examined whether herbivore feeding preferences (i.e., plant palatability) predict herbivore performance (i.e., reflecting plant quality) across relatively small spatial scales, with mixed results. In some studies, feeding preferences predicted performance [18], [19], [20]. In these cases, herbivores were likely choosing plants based on traits (secondary chemicals, amino acid composition, leaf nitrogen, or water content) that directly affected performance [21], [22]. In other studies, a positive correlation between preference and performance was not found [23], [24], [25]. In these cases, herbivores were likely choosing plants based on highly-conservative, simple cues (e.g., avoid food items with an unusual taste), or were choosing foods for reasons unrelated to food quality (e.g., herbivores might select food plants that tended to house few predators). Given these different results, it cannot be assumed that geographic variation in plant palatability necessarily predicts geographic differences in the quality of plants as food for herbivores. This disconnect is important, because understanding geographic variation in plant quality is highly relevant to developing a better understanding of geographic variation in herbivore population dynamics and energy flow through food webs.

In the case of U.S. Atlantic Coast salt marshes, preliminary results suggest that nitrogen, toughness, and secondary chemistry are among the plant traits that vary across latitude and contribute to herbivore preferences for high-latitude plants [14]. Since these traits are likely to directly affect herbivore performance, we speculate that a positive correlation between plant palatability and herbivore performance across latitude is likely to exist in this system. For example, if high-latitude plants have a lower C:N ratio than low-latitude plants, stoichiometric theory would predict that the high-latitude plants would be a nutritionally-superior diet and lead to better herbivore performance [26,27,28,29,30, but see 31]. Similarly, if high-latitude plants are softer than low-latitude plants, optimal foraging studies would predict that herbivores would spend less energy feeding on high-latitude plants, and would digest these plants more easily, and therefore would grow faster on a high-latitude diet [32], [33].

An alternative hypothesis is that herbivores will adapt to perform best on local plants [34,35,36,37,but see 38]. If plant quality varies geographically, this variation should exert selective pressure on herbivores [39]. Across latitude, one thus might expect local adaptation at the regional or site scale. For example, low- and high-latitude herbivores might perform best on low- and high-latitude plants, respectively. However, if plant quality improves continuously toward high latitudes, this might overpower the effects of local adaptation, and herbivores from all latitudes would perform best on high-latitude plants.

We combined field sampling and laboratory experiments to determine whether high-latitude plants (known to be higher in palatability than low-latitude conspecifics) have traits indicating that they should be higher-quality foods for herbivores, and whether differences in these and other traits lead to better herbivore performance, and then whether this latitudinal variation in plant quality outweighs local adaptation of herbivores (if any). In the field, we quantified three plant traits (leaf C:N ratio, chlorophyll content and toughness) across latitude in 3 common salt marsh plant species from 30 sites along the U.S. Atlantic Coast. We further quantified the performance (adult body size) of 2 herbivore species collected from each plant species in the field, to determine whether herbivore performance increased toward higher latitudes in parallel with plant traits. Although a positive correlation across latitude between plant traits and herbivore performance in the field would suggest a causal relationship between these variables, it is also possible that they could be independently responding to environmental factors such as temperature or growing season length [40], [41], [42]. Therefore, we also conducted common-garden experiments to directly test the link between plant origin and herbivore performance. We collected the same 3 plant species and their associated 6 herbivores from the same 30 field sites, which were grouped into 3 broad latitudinal regions (High-, medium-, low-latitude; Table 1). We then conducted factorial experiments (plant region x herbivore region) to examine how herbivore performance might be affected by plant region, herbivore region, and their interaction (i.e., local adaptation).

**Table 1:** Study locations on the Atlantic Coast of the United States.

Region	Location name	State	Latitude	Longitude	NERR or LTER site
High latitude	Harbor Road	ME	43° 19′ 16′′N	70° 34′ 24′′W	Wells NERR
High latitude	Great Bay	NH	43° 02′ 51′′N	70° 50′ 52′′W	Great Bay NERR
High latitude	Nelson Island	MA	42° 44′ 45′′N	70° 49′ 28′′W	PIE LTER
High latitude	Prudence Island	RI	41° 39′ 59′′N	71° 20′ 42′′W	Narragansett Bay NERR
High latitude	Waquoit Bay	MA	41° 34′ 48′′N	70° 31′ 34′′W	Waquoit Bay NERR
Medium latitude	JCR	NJ	39° 32′ 36′′N	74° 19′ 35′′W	Jacques Cousteau NERR
Medium latitude	St. Jones River	DE	39° 05′ 08′′N	75° 27′ 17′′W	Delaware NERR
Medium latitude	Stockton	MD	38° 02′ 26′′N	75° 21′ 56′′W	–
Medium latitude	Red Bank	VA	37° 26′ 50′′N	75° 50′ 03′′W	VCR LTER
Medium latitude	Box Tree	VA	37° 23′ 42′′N	75° 52′ 34′′W	VCR LTER
Low latitude	Masonboro	NC	34° 08′ 28′′N	77° 51′ 50′′W	North Carolina NERR
Low latitude	Goat Island	SC	33° 19′ 54′′N	79° 11′ 54′′W	North Inlet-Winyah Bay NERR
Low latitude	Lighthouse Lane	SC	32° 33′ 17′′N	80° 28′ 09′′W	ACE Basin NERR
Low latitude	Sapelo Island	GA	31° 31′ 04′′N	81° 13′ 54′′W	GCE LTER & Sapelo Island NERR
Low latitude	GTM Reserve	FL	30° 01′ 21′′N	81° 19′ 44′′W	Guana Tolomato Matanzas NERR

Two sites >1 km apart were established at each location for geographic sampling; laboratory experiments used only one of the sites from each location.

In combination, our field and laboratory studies tested two overarching hypotheses:

1. *In the field, leaf C:N and toughness decrease and leaf chlorophyll content increases toward higher latitudes; herbivore performance (body size) increases in parallel with increased plant quality toward higher latitudes.*
2. *In the laboratory, geographic differences in plant quality are more important than local adaptation of herbivores (if any), and hence all herbivore populations will perform best upon higher-latitude plants.*

## Methods

We worked at 11 National Estuarine Research Reserve (NERR) and 3 Long Term Ecological Research (LTER) locations on the U.S. Atlantic Coast, along with one additional location in Stockton, Maryland (Table 1). These study locations covered over 13 degree of latitude, from Florida to Maine, USA. To simplify some comparisons, we divided these 15 locations into 3 regions representing high, medium, and low latitudes (Table 1). This designation is somewhat arbitrary, as the three plant species that we studied have geographic ranges that extend to higher latitudes than our “high latitude” locations, and to lower latitudes than our “low latitude” locations (details below). Although our study locations did not cover the entire latitudinal ranges of these three plant species, they spanned a strong gradient in climate, plant productivity, and plant phenology ([43], [44]. All necessary permits and permissions were obtained for the described field studies, and the studies did not involve endangered or protected species. Permits and permissions were obtained from Wells NERR and Rachel Carson NWR for Harbor Road; Great Bay NERR for Great Bay; PIE LTER for Nelson Island; Narragansett Bay NERR for Prudence Island; Waquoit Bay NERR for Waquoit Bay; Jacques Cousteau NERR for JCR; Delaware NERR for St. Jones River; VCR LTER for Red Bank and Box Tree; North Carolina NERR for Masonboro; North Inlet-Winyah Bay NERR for Goat Island; ACE Basin NERR for Lighthouse Lane; Sapelo Island NERR for Sapelo Island; Guana Tolomato Matanzas NERR for GTM Reserve. The field site of Stockton required no permits as collections were done on public land, and the site was not protected.

We worked with multiple species to help ensure that our results would be general and not idiosyncratic to a single plant or herbivore species. We focused on 3 plant species (*Solidago sempervirens, Iva frutescens, Spartina alterniflora*) and 2 common herbivore species of each (Table 2). The plant species were chosen because they are among the most common in Atlantic Coast salt marshes, represent different growth forms and life-histories, and are tractable for laboratory experiments. The geographic range of *Solidago sempervirens* may extend from the Caribbean (e.g. Bahamas) and Mexico to Eastern Canada (e.g. Quebec, New Brunswick and Newfoundland) [45], [46], [47]. The nominal geographic range of *Iva frutescens* might extend from Florida, USA to the Northeastern Canadian shore (i.e. Nova Scotia) [45], [47], but we rarely observed this species at sites north of Massachusetts. The geographic range of *Spartina alterniflora* reportedly extends from the Caribbean (e.g. Trinidad and Tobago) and Mexico to Canada (e.g. Quebec and Newfoundland) in Northern America, and includes Venezuela, Guyana, Suriname, French Guiana, Brazil, Uruguay, and Argentina in Southern America [45], [47], [48]. As a result, our study locations spanned most of the latitudinal range of *Iva frutescens*, and covered the central part of the latitudinal range of *Solidago sempervirens* and *Spartina alterniflora* on the East Coast of North America. To have a broader representation of herbivore feeding guilds in this study, experimental work on each plant species included one herbivore species from the sucking feeding guild and one from the chewing feeding guild. Species will be referred to generically hereafter, with the exception of the two aphid species in the genus *Uroleucon*.

**Table 2:** Plant and herbivore species studied.

Host plant	Herbivore
*Solidago sempervirens*	*Uroleucon pieloui* (aphid; sucker; specialist [61])
(seaside goldenrod; Asteraceae; herbaceous perennial)	*Paroxya clavuliger* (grasshopper; chewer; generalist [11], [62])
*Iva frutescens*	*Uroleucon ambrosiae* (aphid; sucker; relatively specialized [63])
(marsh-elder; Asteraceae; shrub; perennial)	*Ophraella notulata* (beetle; chewer; specialist [64])
*Spartina alterniflora*	*Prokelisia marginata* (planthopper; sucker; specialist [65])
(smooth cordgrass; Poaceae; clonal perennial)	*Orchelimum fidicinium* (grasshopper; chewer; omnivorous, but *S. alterniflora* as the primary plant diet (personal observations))

Plant common name, family, growth form, herbivore taxonomic group, feeding guild, and diet range are indicated in parentheses.

### Patterns of Leaf Traits and Herbivore Body Size in the Field

To test the hypothesis that leaf C:N and toughness would decrease and leaf chlorophyll content and herbivore body size would increase toward higher latitudes, we sampled plant traits and herbivore body size in the summers of four years (2004–2007). We sampled leaf chlorophyll content and toughness in 2004 (*Solidago*), 2005 (*Iva*) and 2006 (*Spartina*). Herbivores were collected for body size measurements in the same year that their host plant traits were measured, except that we collected *Paroxya* (*Solidago*) and *Prokelisia* (*Spartina*) in 2007 for logistical reasons. Two separate sites (>1 km apart) were established at each location, to give a total of 30 study sites; however, not all species were present at each site, reducing final sample sizes. At each site, we measured leaf chlorophyll content (a unitless estimate of nitrogen content) and toughness (grams required to break leaf tissue), using a portable chlorophyll meter and penetrometer on 3 leaves per plant. Six plants per species were randomly sampled at each site, and results averaged over leaves and plants to yield a single value for each site. We did not measure *Spartina* chlorophyll because its leaves were too narrow to obtain a reading with the chlorophyll meter. We collected leaves for analyzing leaf C and N content in July 2006 and July 2007. At each site, we collected a single leaf from each of 6 plants of each species. Leaves were lyophilized, pulverized, and sent to the University of Georgia Chemical Analysis laboratory for analysis. Data were averaged across plants within a site, and across years (no significant variation among years based on ANCOVA, with site latitude as covariate), to yield a single value for each site. Body size of insects is sometimes measured directly but in other cases (when individuals adopt variable postures after being preserved or pinned) is best estimated by measuring tibia length. Both body size or tibia length of insects has been reportedly associated with insect performance or fitness [49], [50], [51]. We reanalyzed published body size (tibia length) data for *Orchelimum* collected from the same or nearby sites [52]. We collected the other five herbivore species (n = 4 to 20/species/site) ourselves, preserved them in 70% alcohol, and measured them under a dissecting microscope. We measured body length of *Prokelisia* and tibia length of the other 4 species to a precision of 0.01 mm. Body size data for *Prokelisia* and *Orchelimum* were previously published [30] but are included here for completeness. Data on variation in plant traits and herbivore body size were regressed against latitude (with each site treated as a single point), using the Statistix program. We tested for non-linear patterns by assessing the significance of the polynomial term.

### Herbivore Performance

To test the hypothesis that geographic differences in plant quality are more important than local adaptation of herbivores (if any), and thus that all herbivore populations will perform best upon higher-latitude plants, we sought to conduct three by three factorial growth experiments, crossing host plants collected from three geographic regions with herbivores collected from three geographic regions. However, for logistical reasons (usually because some herbivores were rare and thus hard to collect in some geographic regions), the *Solidago-U. pieloui* and *Solidago-Paroxya* experiments had a 2 (plant regions) × 2 (herbivore regions) design, and the *Spartina-Orchelimum* experiment had a 3 (plant regions) × 2 (herbivore regions) design. Each combination of plant x herbivore regions had at least 5 replicates.

Seeds (*Solidago* and *Iva*) or clonal ramets (*Spartina*) were collected from 15 sites (one site from each location, Table 1), except that *Iva* didn’t occur at the Wells and Great Bay NERRs, and therefore its seeds were collected from two additional sites within the same region (Bluff Point: 41° 19′ 36′′N, 72° 02′ 06′′W; Sheep Pen: 41° 38′ 59′′N, 71° 20′ 49′′W). We propagated plants in a mixture of 60% potting soil and 40% sand in the greenhouse at the University of Georgia Marine Institute. Herbivores were collected from the same 15 sites, and host quality assessed by growing herbivores on ad-lib plant leaves or whole plants. In most cases, due to the life cycle of the herbivores, experiments started in late June. The aphids *U. pieloui* and *U. ambrosiae* and the planthopper *Prokelisia* were collected as adults from the field, laboratory stock colonies established on caged plants collected from the same sites as the herbivores, and population (aphids) or individual (planthoppers) growth followed for 4 and 6 weeks, respectively, after inoculating caged experimental plants with 5 and 3 juveniles respectively. We recorded aphid population size weekly, and we removed planthopper individuals that had reached adulthood twice a week and measured their body length under a dissecting microscope. The grasshoppers *Paroxya* and *Orchelimum* were collected as nymphs from the field, raised individually in the laboratory in jars with fresh leaves (replaced every 2 days), and measured (relative growth rate of body mass) over 1 month. The beetle *Ophraella* was collected as gravid females that were allowed to lay eggs in the laboratory. Upon hatching, beetle larvae were housed individually in Petri dishes, supplied with fresh leaves every 2 days, and growth (relative growth rate of larval length) assessed every 2 days until pupation. Herbivores that were raised in jars or Petri dishes were kept in an air-conditioned room at 27.5°C. Herbivores raised on caged plants were kept in the greenhouse at the University of Georgia Marine Institute at ambient conditions. The room and greenhouse are both subject to natural lighting; lighting inside the room was supplemented during the day with fluorescent lighting. The temperature conditions in the air-conditioned room likely fell in between conditions at our high- and low-latitude field sites; temperature conditions in the greenhouse were likely similar to those at low-latitude field sites. We analyzed most herbivore performance data using 2-way ANOVA, with plant region and herbivore region as main effects. For experiments with *U. pieloui* (on *Solidago* plants) and *U. ambrosiae* (on *Iva* plants), we analyzed population growth data with repeated-measures ANOVA. The independent and dependent variables were summarized in Table 3.

**Table 3:** The independent and dependent variables for the study of herbivore performance.

Independent variables	Dependent variables
***–Solidago*** ** plants and the herbivore ** ***U. pieloui–***	
Week, Plant region, Herbivore region	*U. pieloui* population
***–Solidago*** ** plants and the herbivore ** ***Paroxya–***	
Plant region, Herbivore region	*Paroxya* body mass, survivorship
***–Iva*** ** plants and the herbivore ** ***U. ambrosiae–***	
Week, Plant region, Herbivore region	*U. ambrosiae* population
***–Iva*** ** plants and the herbivore ** ***Ophraella–***	
Plant region, Herbivore region	*Ophraella* larval body size, larval period, survivorship
***–Spartina*** ** plants and the herbivore ** ***Prokelisia–***	
Plant region, Herbivore region	*Prokelisia* adult body size, survivorship
***–Spartina*** ** plants and the herbivore ** ***Orchelimum–***	
Plant region, Herbivore region	*Orchelimum* body mass, survivorship

## Results

### Patterns of Leaf Traits and Herbivore Body Size in the Field

**Figure 2:**
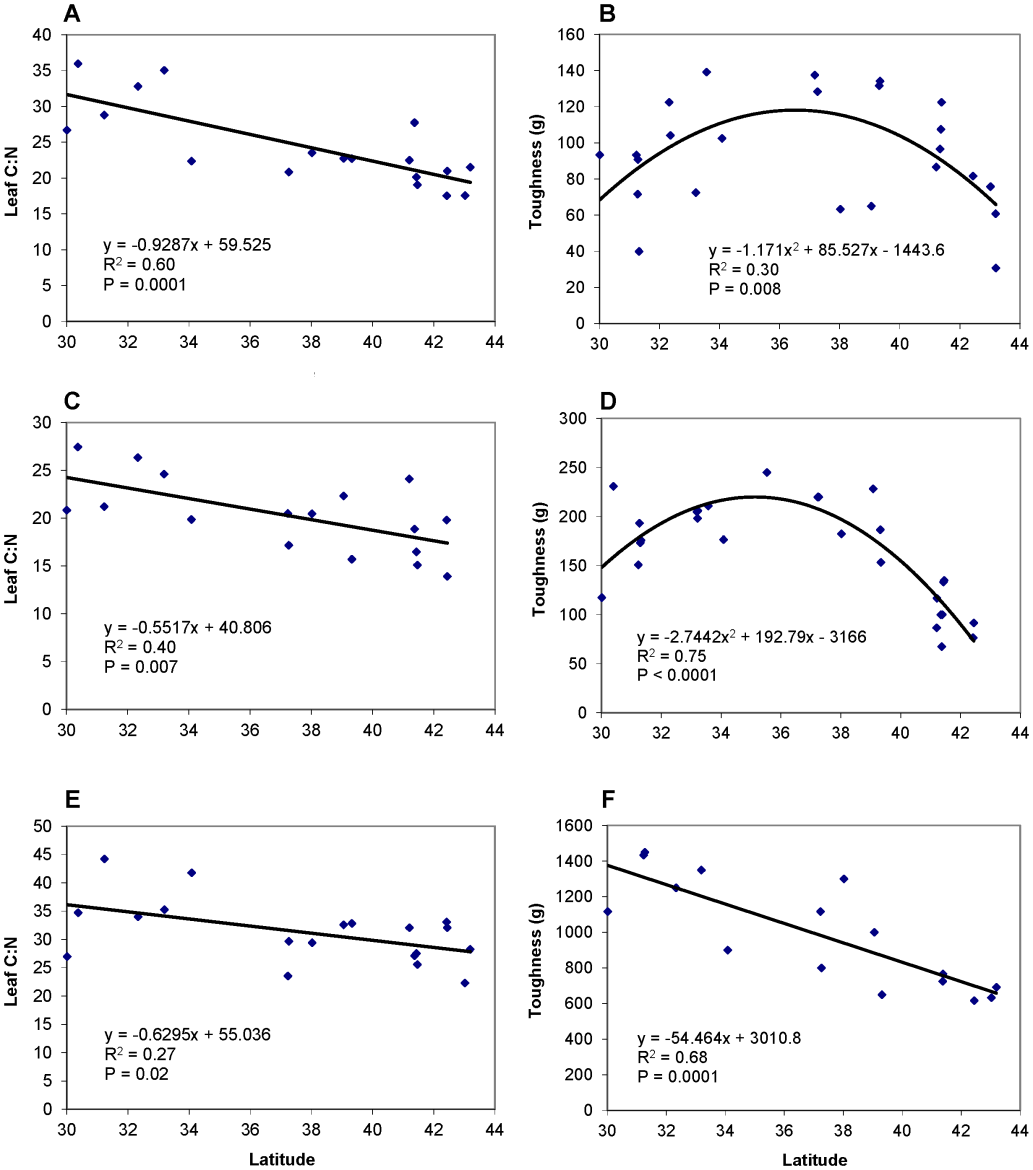
Patterns of plant traits in the field. Leaf C:N and toughness of *Solidago sempervirens* (A, B), *Iva frutescens* (C, D) and *Spartina alterniflora* (E, F) versus latitude. Each point represents a single study site.

Most plant traits changed in ways that would suggest that high-latitude plants were a higher quality food than low-latitude plants for herbivores. Leaf chlorophyll content, an indicator of leaf nitrogen content, increased toward higher latitudes in both *Solidago* and *Iva* (P = 0.003 and 0.04 respectively; chlorophyll content was not measured in *Spartina*). Leaf C content did not vary across latitude in all three plant species, but leaf N content increased toward higher latitudes in all three species (P = 0.007, 0.0001 and 0.01 for *Solidago*, *Iva* and *Spartina* respectively). As a result, leaf C:N decreased toward high latitudes in all 3 plant species (Fig. 2A, C, E). Leaf toughness decreased toward higher latitudes in *Spartina* (Fig. 2F), but showed a hump-shape relation with latitude in *Solidago* and *Iva* (Fig. 2B, D).

**Figure 3:**
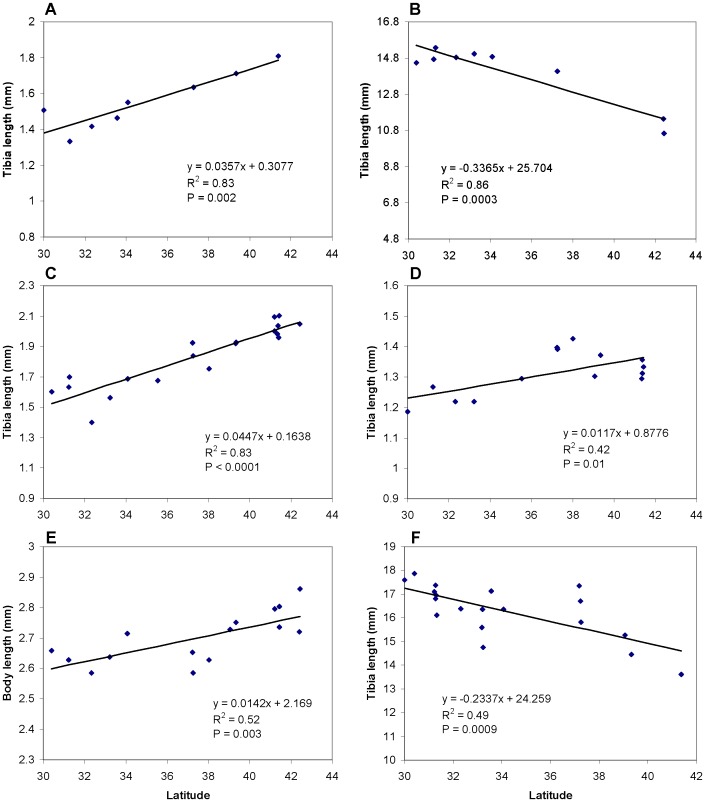
Patterns of herbivore body size in the field. Latitudinal variations in body size of *U. pieloui* and *Paroxya* on *Solidago* plants (A, B), *U. ambrosiae* and *Ophraella* on *Iva* plants (C, D), and *Prokelisia* and *Orchelimum* on *Spartina* plants (E, F). Each point represents a single study site.

Herbivores were larger at high latitudes in 4 out of 6 cases. Tibia length or body length increased toward higher latitudes in *U. pieloui*, *U. ambrosiae*, *Ophraella*, and *Prokelisia* (Fig. 3A, C, D, E). In contrast, the grasshoppers *Paroxya* and *Orchelimum* were smaller at higher latitudes (Fig. 3B, F).

### Herbivore Performance

#### The impact of plant region on herbivore performance

**Figure 4:**
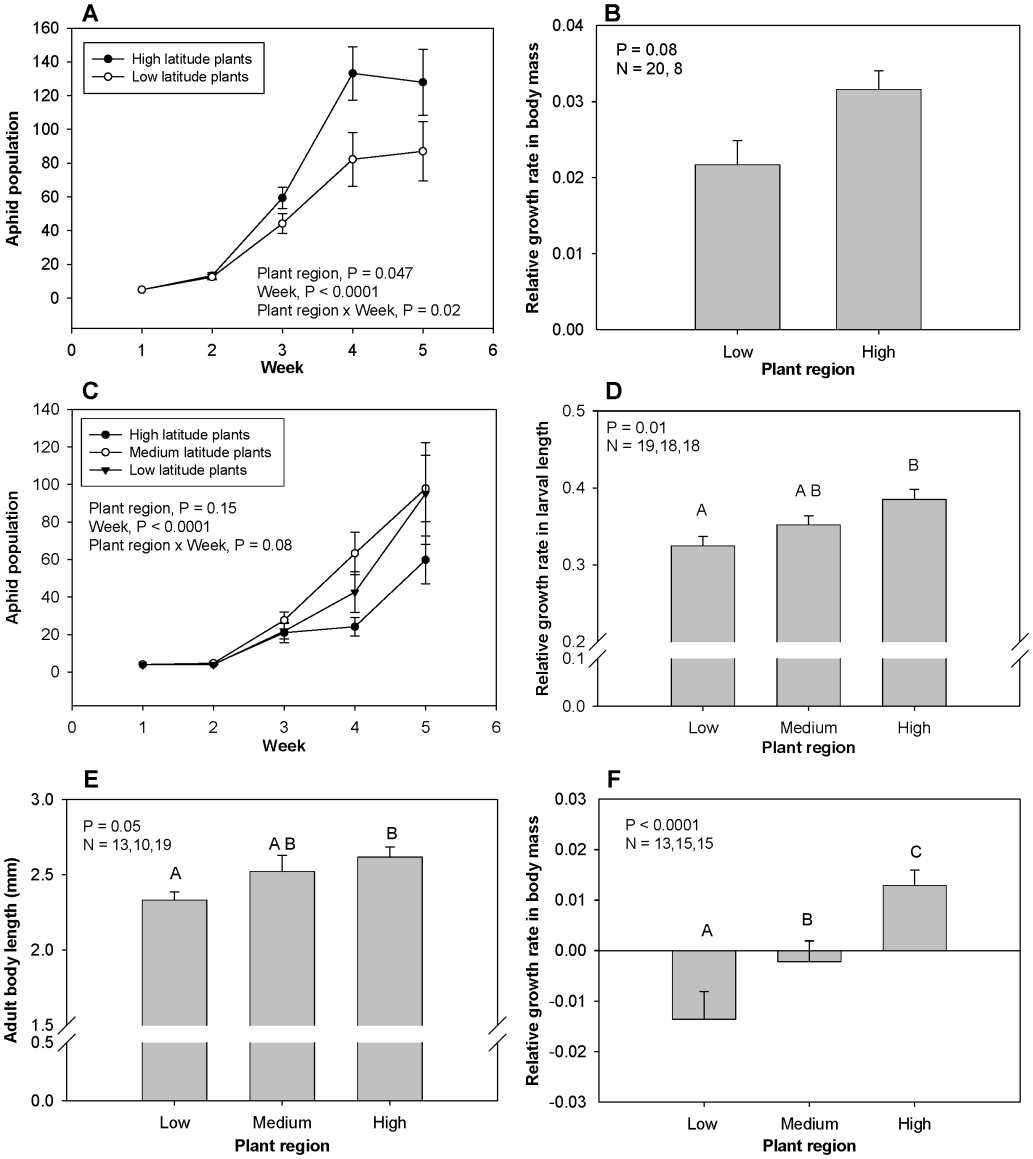
The impact of plant region on herbivore performance. The impact of *Solidago* plant regions on *U. pieloui* population growth and *Paroxya* growth rate (A, B); the impact of *Iva* plant regions on *U. ambrosiae* population growth and *Ophraella* growth rate (C, D); the impact of *Spartina* plant regions on *Prokelisia* body size and *Orchelimum* growth rate (E, F).

Plant collected from higher latitudes supported better herbivore performance in 4 out of 6 herbivore species, with a fifth species showing a trend (P = 0.08) towards better growth on high-latitude plants. *Solidago* plants originating from high latitudes supported higher population growth rates of the aphid *U. pieloui* (Fig. 4A) and tended to support faster body-mass growth of the grasshopper *Paroxya* (P = 0.08) (Fig. 4B), regardless of herbivore region (no plant origin by herbivore region interactions). *Iva* plant region did not affect population growth of the aphid *U. ambrosiae* (Fig. 4C); however, *Iva* plants originating from high latitudes supported faster body-size growth of larvae of the beetle *Ophraella* (Fig. 4D), a shorter larval period (P = 0.008), and higher survivorship to adulthood (P = 0.006) regardless of herbivore region (no plant origin by herbivore region interactions). *Spartina* plants originating from high latitudes supported greater body size (Fig. 4E) and tended to support better survivorship (P = 0.06) of the planthopper *Prokelisia*, and also supported faster body-mass growth (Fig. 4F) and better survivorship (P<0.0001) of the grasshopper *Orchelimum* regardless of herbivore region. Details of statistical results are provided in Appendix S1.

#### The impact of herbivore region on herbivore performance

**Figure 5:**
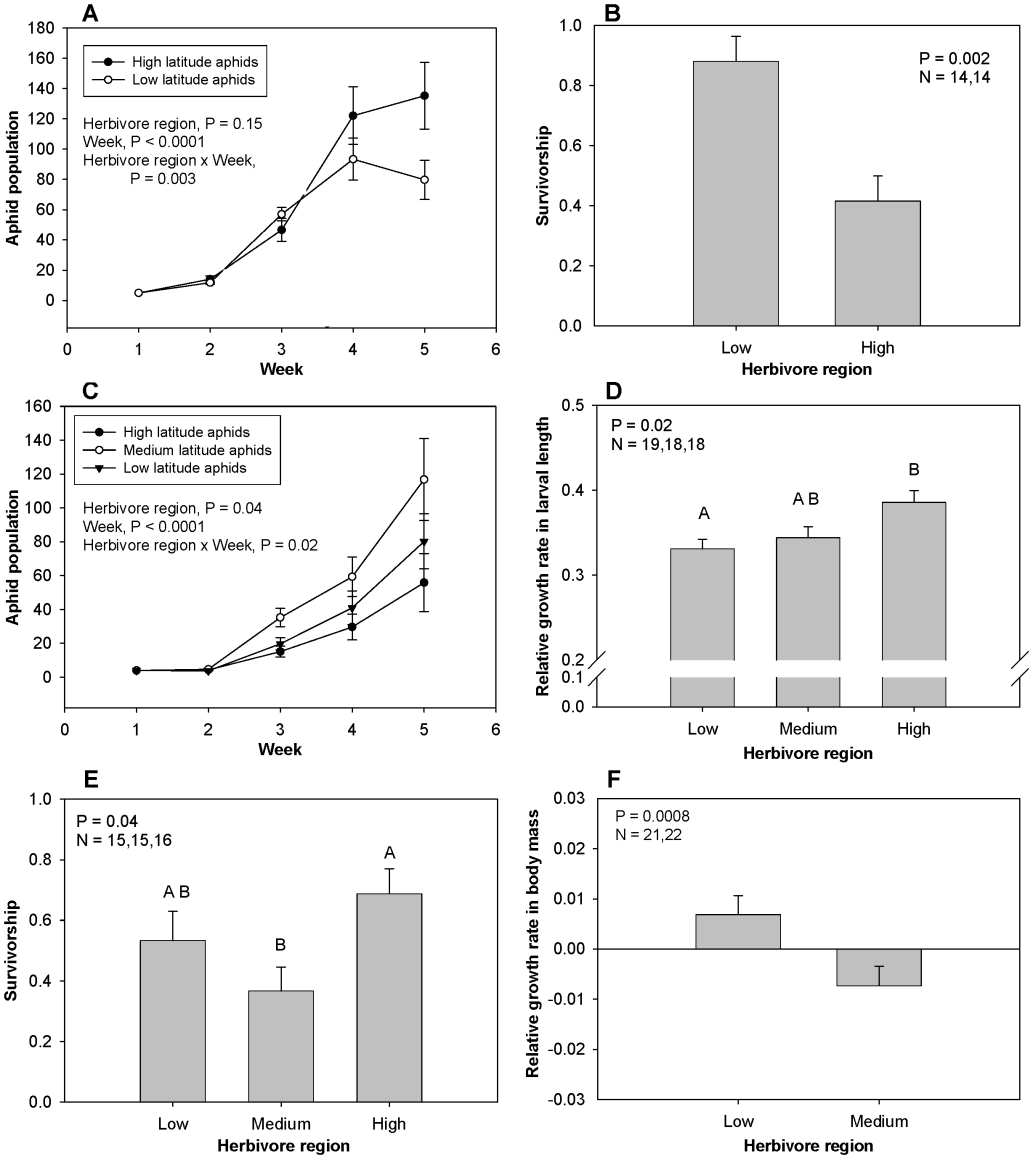
The impact of herbivore region on herbivore performance. The impact of herbivore regions on A) *U. pieloui* population growth, B) *Paroxya* survivorship, C) *U. ambrosiae* population growth, D) *Opraella* larval growth rate, E) *Prokelisia* survivorship, and F) *Orchelimum* growth rate.

The geographic region from which herbivores were collected affected herbivore performance in all six species, but the nature of the relationship differed among species. Of the 6 herbivore species, high-latitude individuals performed better in 2 species, medium-latitude individuals better in 1 species but worse in another species, and low-latitude individuals better in 2 species. In no cases were there any interactions between plant and herbivore regions that would have indicated local adaptation at the geographic scale. For *Solidago* herbivores, populations of *U. pieloui* from high latitudes grew faster than those from low latitudes, with differences most notable at the end of experiment (Fig. 5A); in contrast, although geographic region did not affect *Paroxya* individual growth rate (P = 0.72), individuals from low latitudes had a higher survivorship than those from high latitudes (Fig. 5B). For *Iva* herbivores, populations of *U. ambrosiae* from medium latitudes grew fastest (Fig. 5C); in contrast, *Ophraella* from high latitudes had a better individual growth rate than did individuals from low latitudes (Fig. 5D), a shorter larval period (P = 0.02) and bigger larval size (P = 0.02). For *Spartina* herbivores, populations of *Prokelisia* originating from different regions differed in survivorship (medium latitudes had lowest survivorship; Fig. 5E), but did not differ in adult size (P = 0.54); whereas *Orchelimum* originating from low latitudes had a better individual growth rate (Fig. 5F) and tended to have better survivorship (P = 0.06). Details of statistical results are provided in Appendix S1.

## Discussion

Our results for plant traits (especially leaf N content and C:N ratio) suggested that high-latitude plants (known to be higher in palatability than low-latitude conspecifics) should be of higher quality to herbivores. Consistent with this prediction, herbivore body size increased at high latitudes for four of six herbivore species, and likely was precluded from doing so by life-history constraints in the other two species. In the greenhouse, high-latitude plants supported better performance of herbivores in four, or possibly five, of six cases. Although the region from which herbivores were collected also affected herbivore performance, patterns differed across species and herbivore region never interacted with plant region, indicating a lack of local adaptation at this spatial scale.

The quality of plants to herbivores is likely affected by many plant traits. In this study system, Siska et al. [14] previously conducted preliminary comparisons of nitrogen, toughness and chemistry and found that nitrogen content was higher in high- versus low-latitude plants for *Spartina*, and possibly for *Solidago*, but did not differ for *Iva*; that leaf toughness was lower in high- versus low-latitude plants for *Spartina* but not for *Iva* or *Solidago*; and that crude extracts from high latitude plants were more palatable than those from low-latitude plants for *Iva* and *Spartina*, but not *Solidago*. We collected more extensive data than Siska et al. [14] on leaf nitrogen content and leaf toughness, sampling across the entire latitudinal gradient rather than only at the extremes. Our data indicate that leaf C:N declines linearly in all three species with increasing latitude, a pattern that was driven primarily by increases in leaf N, because leaf C did not vary significantly across latitude. Thus, in contrast to Siska et al. [14], our more extensive data indicates that all three plant species are more nutritious at high versus low latitudes. These results are consistent with worldwide patterns of increasing leaf N at high latitudes across several thousand plant species [15]. Leaf toughness decreased linearly with increasing latitude for *Spartina*, consistent with the findings of Siska et al. [14], but showed a hump-shaped relationship with latitude for *Solidago* and *Iva*, likely explaining why Siska et al. [14] found no difference in toughness of these two species when they compared high versus low latitude sites. We did not measure leaf secondary chemistry.

In combination with results from Siska et al. [14], these results suggest that all three plant species should be higher in quality for herbivores at high latitudes. *Solidago* has a higher nitrogen content at high versus low latitudes, *Iva* has a higher nitrogen content and more palatable extracts, and *Spartina* has a higher nitrogen content, more palatable extracts (likely driven by lower phenolic content [14]) and is softer. The trends in our study (including the hump-shaped patterns of leaf toughness for *Solidago* and *Iva*) were continuous rather than step functions, indicating that they are not primarily driven by the fairly abrupt latitudinal transition between geographic regions that experience hard winter freezes and those that do not, a border that is associated with differences in winter standing biomass, leaf litter, and herbivore community composition [43], [52]. While our field survey covered a considerable portion of latitudinal ranges of the focal plant species, their ranges may extend further north along the Northeastern Canadian shore, and it would be interesting to investigate how the plant traits measured in this study (e.g. Fig. 2B) would change at these higher latitudes. We are reluctant to speculate, since a plant trait could be a function of many factors. For example, plant toughness is related to multiple biotic (leaf life span, growth rate, herbivory) and abiotic factors (light intensity, physical disturbance) [12], [14], [53], [54].

Four of the six herbivores increased in body size towards higher latitudes, consistent with a recent hypothesis that high-quality plants at high latitudes might help explain Bergmann’s rule in herbivores [30]. The two exceptions were the two grasshopper species, which decreased in body size towards high latitudes. This result was probably driven by the constraints imposed on large, univoltine species by the short growing season at high latitudes. In short, at high latitudes these species may run out of the time needed in order to grow large [30], [52]. This conclusion is supported by the fact that, although they were smaller at high latitudes in the field, both grasshopper species grew better in the laboratory when fed high- versus low-latitude plants.

Because many factors vary across latitude along with plant quality, it was necessary to corroborate the correlation between plant quality and herbivore performance observed in the field with a common-garden laboratory experiment. The results of this study strongly supported the hypothesis that geographic variation in herbivore performance is affected by variation in plant quality across latitude, which in turn is correlated with plant palatability. Five out 6 herbivores performed significantly or marginally significantly (P = 0.08) better on plants from higher latitudes. The only exception was *U. ambrosiae*, which performed similarly on plants from all three regions. Past studies in Atlantic Coast salt marshes determined that herbivores prefer to eat high- versus low-latitude plants, and that latitudinal variation in plant palatability and relevant traits are in part constitutive and can continue to exist in a common garden [9], [11], [14]; we can now conclude that, in this system, herbivore preference predicts herbivore performance at geographic scales. Latitudinal variation in herbivore preference has also been documented in other study systems [8], [13]. If the relationship between geographic variation in herbivore preference and performance is general, then geographic differences in plant palatability may not just affect what herbivores eat, but also may affect individual growth rates, population dynamics, and even trophic structure through species interactions.

The region from which herbivores were collected significantly affected individual or population growth rate in all 6 herbivore species in laboratory experiments, but the pattern was mixed. Of the 6 herbivore species, high latitude individuals performed better in 2 cases (1 sucking and 1 chewing herbivore), medium latitude individuals better in 1 case (sucking herbivore) but worse in another case (sucking herbivore), and low latitude individuals better in 2 cases (2 chewing herbivores). Therefore, although herbivore origin affected herbivore performance, the pattern was not general across herbivores or simple with respect to latitude, nor did insect feeding guild (i.e. sucking vs. chewing) help explain the results.

In this study, plant quality increased with increasing latitudes and in most cases supported better herbivore performance at high latitudes. But field sampling and reciprocal transplant experiments have shown that herbivore pressure is stronger at low latitudes in this system [12]. Why doesn’t better herbivore performance at high latitudes produce higher herbivore population densities and feeding pressure at high latitudes? Some factor(s) must stop herbivores at high latitudes from producing larger populations that would create stronger feeding pressures on high-latitude plants. Although we did not explore this issue in the current study, the most likely explanation is that the shorter summers and more severe winters at high latitudes reduce the length of the herbivore growing season and the survivorship of herbivores at high latitudes. For example, multi-voltine species usually have fewer generations at high latitudes [55], [56], and severe winters at high latitudes often reduce overwintering survivorship [57], [58], [59].

Herbivores often show local adaptation to varying host plants [34], [35], [36], [37], but we found no evidence of local adaptation at the geographic scale. The lack of local adaptation at this scale is probably because latitudinal variation in plant quality is so strong that it essentially precludes the possibility of local adaptation–high-latitude plants are not just “different” from low-latitude plants, in the way that one population of a plant might deploy a different array of secondary metabolites than another, but are simply superior as a diet.

Maintaining biodiversity is an important issue for habitat management. When considering the importance of biological diversity, scientists are increasingly considering not just species richness, but also with the role of genetic diversity within species. The importance of this concern is supported by this study, in which both plant region and herbivore region of origin strongly affected the performance of herbivores feeding on plants. Our results demonstrated that populations of both plants and herbivores from different regions have different ecological traits, and therefore suggests that this regional variation must be taken into account in order to understand and manage plant-herbivore interactions in any particular region [35]. For example, coastal managers are faced with predicting the consequences that global change is likely to have for salt marshes. An important type of effect that is likely to be produced by global change is a “re-shuffling” of biotas, which may produce local assemblages comprised of populations or species that lack a history of association [60]. Although the impacts of re-shuffling are still largely unclear, our results show that they are likely to affect both individual and population growth rates of herbivores, and thus likely also to affect top-down control of plants by herbivores.

This study adds to a growing body of work showing that the nature of species interactions varies geographically [4]. Studying geographic variation in interactions is important because it can reveal how ecological processes at any local site are shaped by broader factors that operate across geographic regions. In order to develop general theories of ecology or evolution, scientists must understand this geographic variation and incorporate it into their models.

## Supporting Information

Appendix S1 **Summary of ANOVA results for the effects of plant regions and herbivore regions on herbivore performance.**
(DOC)
