# Supplementary material for: Preference and Performance in Plant–Herbivore Interactions across Latitude–A Study in U.S. Atlantic Salt Marshes
Source: PLoS One. 2013 Mar 22;8(3):e59829. doi: 10.1371/journal.pone.0059829 (PMC3606276; doi:10.1371/journal.pone.0059829)
Supplement: Appendix S1 — Summary of ANOVA results for the effects of plant regions and herbivore regions on herbivore performance. (DOC) [file pone.0059829.s001.doc]

**Appendix S1**

**a)** Summary of ANOVA results for the effects of *Solidago* plant regions and herbivore regions on herbivore performance. RGR = relative growth rate. Significant *P*-values (critical level = 0.05) are in bold.

*Solidago* plants and the herbivore *U. pieloui*

Repeated-measures ANOVA on *U. pieloui* population over weeks

| **Source** | **df** | ***F*** | ***P*** |
| --- | --- | --- | --- |
| **--Within subjects--** |  |  |  |
| Week | 4 | 57.17 | **<0.0001** |
| Week*Plant region | 4 | 3.17 | **0.02** |
| Week*Herbivore region | 4 | 4.18 | **0.003** |
| Week*Plant region*Herbivore region | 4 | 0.11 | 0.98 |
| Error | 128 |  |  |
| **--Between subjects--** |  |  |  |
| Plant region | 1 | 4.27 | **0.047** |
| Herbivore region | 1 | 2.13 | 0.15 |
| Plant region*Herbivore region | 1 | 0.03 | 0.87 |
| Error | 32 |  |  |

*Solidago* plants and the herbivore *Paroxya*

Two-way ANOVA on *Paroxya* performance

| **Source** | **df** | ***F*** | ***P*** |
| --- | --- | --- | --- |
| **--*Paroxya* body mass (RGR)--** |  |  |  |
| Plant region | 1 | 3.29 | 0.08 |
| Herbivore region | 1 | 0.14 | 0.72 |
| Plant region*Herbivore region | 1 | 0.21 | 0.65 |
| Error | 24 |  |  |
| **--*Paroxya* survivorship--** |  |  |  |
| Plant region | 1 | 2.41 | 0.13 |
| Herbivore region | 1 | 12.22 | **0.002** |
| Plant region*Herbivore region | 1 | 0.07 | 0.80 |
| Error | 24 |  |  |

**b)** Summary of ANOVA results for the effects of *Iva* plant regions and herbivore regions on herbivore performance. RGR = relative growth rate. Significant *P*-values (critical level = 0.05) are in bold.

*Iva* plants and the herbivore *U. ambrosiae*

Repeated-measures ANOVA on *U. ambrosiae* population over weeks

| **Source** | **df** | ***F*** | ***P*** |
| --- | --- | --- | --- |
| **--Within subjects--** |  |  |  |
| Week | 4 | 50.47 | **<0.0001** |
| Week*Plant region | 8 | 1.80 | 0.08 |
| Week*Herbivore region | 8 | 2.40 | **0.02** |
| Week*Plant region*Herbivore region | 16 | 1.85 | **0.03** |
| Error | 144 |  |  |
| **--Between subjects--** |  |  |  |
| Plant region | 2 | 2.01 | 0.15 |
| Herbivore region | 2 | 3.47 | **0.04** |
| Plant region*Herbivore region | 4 | 1.40 | 0.25 |
| Error | 36 |  |  |

*Iva* plants and the herbivore *Ophraella*

Two-way ANOVA on *Ophraella* performance

| **Source** | **df** | ***F*** | ***P*** |
| --- | --- | --- | --- |
| **--*Ophraella* larval body size--** |  |  |  |
| Plant region | 2 | 2.24 | 0.12 |
| Herbivore region | 2 | 4.55 | **0.02** |
| Plant region*Herbivore region | 4 | 0.96 | 0.44 |
| Error | 46 |  |  |
| **--*Ophraella* larval period--** |  |  |  |
| Plant region | 2 | 5.34 | **0.008** |
| Herbivore region | 2 | 4.23 | **0.02** |
| Plant region*Herbivore region | 4 | 0.86 | 0.50 |
| Error | 46 |  |  |
| **--*Ophraella* larval body size (RGR)--** |  |  |  |
| Plant region | 2 | 4.80 | **0.01** |
| Herbivore region | 2 | 4.19 | **0.02** |
| Plant region*Herbivore region | 4 | 0.51 | 0.73 |
| Error | 46 |  |  |
| **--*Ophraella* survivorship to adulthood--** |  |  |  |
| Plant region | 2 | 5.82 | **0.006** |
| Herbivore region | 2 | 0.95 | 0.39 |
| Plant region*Herbivore region | 4 | 1.55 | 0.20 |
| Error | 48 |  |  |

**c)** Summary of ANOVA results for the effects of *Spartina* plant regions and herbivore regions on herbivore performance. RGR = relative growth rate. Significant *P*-values (critical level = 0.05) are in bold.

*Spartina* plants and the herbivore *Prokelisia*

Two-way ANOVA on *Prokelisia* performance

| **Source** | **df** | ***F*** | ***P*** |
| --- | --- | --- | --- |
| **--*Prokelisia* adult body size--** |  |  |  |
| Plant region | 2 | 3.19 | **0.05** |
| Herbivore region | 2 | 0.64 | 0.54 |
| Plant region*Herbivore region | 4 | 2.24 | 0.09 |
| Error | 33 |  |  |
| **--*Prokelisia* survivorship--** |  |  |  |
| Plant region | 2 | 3.09 | 0.06 |
| Herbivore region | 2 | 3.48 | **0.04** |
| Plant region*Herbivore region | 4 | 0.69 | 0.61 |
| Error | 37 |  |  |

*Spartina* plants and the herbivore *Orchelimum*

Two-way ANOVA on *Orchelimum* performance

| **Source** | **df** | ***F*** | ***P*** |
| --- | --- | --- | --- |
| **--*Orchelimum* body mass (RGR)--** |  |  |  |
| Plant region | 2 | 14.24 | **< 0.0001** |
| Herbivore region | 1 | 13.26 | **0.0008** |
| Plant region*Herbivore region | 2 | 1.40 | 0.26 |
| Error | 37 |  |  |
| **--*Orchelimum* survivorship--** |  |  |  |
| Plant region | 2 | 28.92 | **< 0.0001** |
| Herbivore region | 1 | 3.83 | 0.06 |
| Plant region*Herbivore region | 2 | 0.94 | 0.40 |
| Error | 39 |  |  |
